# Sertoli cell ablation and replacement of the spermatogonial niche in mouse

**DOI:** 10.1038/s41467-019-13879-8

**Published:** 2020-01-02

**Authors:** Tetsuhiro Yokonishi, Jennifer McKey, Shintaro Ide, Blanche Capel

**Affiliations:** 1 Department of Cell Biology, Duke University Medical Center, Durham, NC 27710 USA; 2 Department of Urology, Yokohama City University, Yokohama, Japan; 3 Division of Nephrology, Department of Medicine, Duke University School of Medicine, Durham, NC 27710 USA

**Keywords:** Regeneration, Stem-cell niche

## Abstract

Spermatogonia, which produce sperm throughout the male lifetime, are regulated inside a niche composed of Sertoli cells, and other testis cell types. Defects in Sertoli cells often lead to infertility, but replacement of defective cells has been limited by the inability to deplete the existing population. Here, we use an FDA-approved non-toxic drug, benzalkonium chloride (BC), to deplete testis cell types in vivo. Four days after BC administration, Sertoli cells are preferentially depleted, and can be replaced to promote spermatogenesis from surviving (host) spermatogonia. Seven days after BC treatment, multiple cell types can be engrafted from fresh or cryopreserved testicular cells, leading to complete spermatogenesis from donor cells. These methods will be valuable for investigation of niche-supporting cell interactions, have the potential to lead to a therapy for idiopathic male infertility in the clinic, and could open the door to production of sperm from other species in the mouse.

## Abstract (web summary)

Sertoli cells and other somatic cells of the testis comprise the germ cell niche and are critical to regulate spermatogenesis. Here the authors present a method in which Sertoli cells are selectively targeted for ablation by the compound benzalkonium chloride (BC) in mice, and the spermatogenic niche is subsequently repopulated in regions that have been affected by BC treatment.

## Introduction

The efficient production of sperm from spermatogonia requires the coordinated interplay between germ cells and various somatic support cells making up the spermatogenic niche. Approximately 1:100 adult males is azoospermic, and in ~40% of these cases the defect is idiopathic and may lie with the germ cell itself or any of the somatic cell types making up the spermatogenic niche. Of these cell types, Sertoli cells reside within the testis tubules attached to the same basal lamina as the spermatogonia, while Leydig cells, peritubular myoid cells (PMCs) and macrophages lie immediately outside the tubules in the supporting stroma. Since the Sertoli cells interact directly with germ cells and are vital to provide morphological and nutritional support for spermatogenesis, their dysfunction is often the cause of spermatogenic failure^1–5^.

To compensate for Sertoli cell dysfunction, several therapeutic approaches have been investigated, such as transplantation of healthy Sertoli cells^6^, supplementation of missing growth factors by viral transduction^7–10^, or explant and culture of testis tissue in vitro^11^. Although researchers showed evidence that offspring did not carry the transgene^7–10^, it is difficult to exclude the possibility of viral transmission to the next generation, and no human testis culture system has yet been established. Since auto-transplantation of testicular cells from Hodgkin’s lymphoma patients was reported^12,13^, Sertoli cell transplantation has been a possible approach for the clinic. However, long-term success depends upon sufficient space being made available along the basal lamina for repopulation of the tubules by donor Sertoli cells. For in vivo feasibility studies, some groups have used transgenic mouse models expressing cytotoxic genes or specific toxin receptors to eliminate Sertoli cells prior to transplantation^14^. However, since these approaches require genetic manipulations, they are not suitable for clinical studies. Other studies used cadmium injection into the testis to deplete Sertoli cells, but cadmium administration has many adverse side effects and could not be used in the clinic^15^.

Here, we demonstrate the use of a well-described FDA-approved nontoxic drug, benzalkonium chloride (BC), to remove host Sertoli cells and enable cell transplantation into the testis in vivo. BC is a quaternary ammonium that acts as a detergent to disrupt the lipid membrane of cells. Originally developed as a germicide to kill microorganisms, it was first used in the ophthalmic industry in the 1940s as a preservative in hard contact lens solutions.

We show that treatment of the testis with BC leads to rapid depletion of Sertoli cells. We describe two windows of opportunity for engraftment of various testis cell types after BC injection into the rete testis. Within 4 days of administration, BC preferentially depletes only Sertoli cells, leaving a scaffold into which healthy, isolated Sertoli cells can be engrafted to allow rescue of spermatogenesis from surviving (host) spermatogonia. At later times after BC treatment, some germ cell and stromal support cell types are also lost, most likely secondary to the loss of the trophic support of Sertoli cells. Significantly, these cells can be replaced by engraftment of fresh or cryopreserved testicular cells, leading to recovery of spermatogenesis from donor cells.

We show that the effects of BC extend to a large animal model (canine). As BC is a common component of eye drops, we anticipate that our Sertoli/niche cell ablation method could be safely adapted for the clinic to overcome the weaknesses of previous approaches. We anticipate that this method could be used to rescue host fertility, and possibly to produce xenogeneic sperm in mouse. This approach will also open up opportunities for in vivo studies of niche cell interactions and function.

## Results

### Neonatal Sertoli cell selective depletion in vitro

**Fig. 1:**
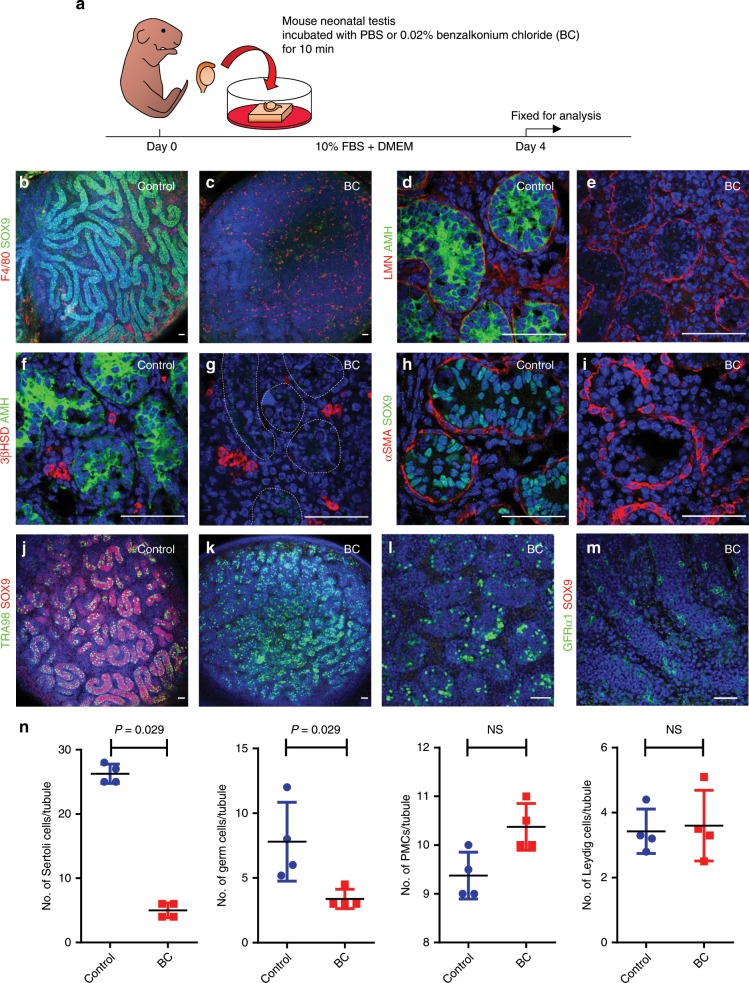
BC treatment eliminates neonatal Sertoli cells in cultured testes. **a** Schematic representation of experimental procedures for in vitro Sertoli cell ablation. **b**–**m** In all, 1.5 dpp mouse testis cultured for 4 days following incubation with **b**, **d**, **f**, **h**, **j** PBS or **c**, **e**, **g**, **i**, **k**–**m** 0.02% BC for 10 min. Samples were stained with antibodies against **b** and **c** SOX9 (green), F4/80 (red); **d**, **e** AMH (green), LMN (red); **f**, **g** AMH (green) and 3βHSD (red). Hoechst (blue) was used to stain nuclei. Expected seminiferous cord domain is marked by dotted lines. **h**, **i** SOX9 (green) and αSMA (red). **j**–**l** SOX9 (red) and TRA98 (green). **m** SOX9 (red) and GFRα1 (green). Ten independent experiments. Scale bar: 50 µm. **n** After treatment with 0.02% BC, numbers of Sertoli cells and germ cells/tubule were reduced relative to untreated controls; however, counts of residual Leydig and myoid cells were similar to untreated controls. Data were analyzed from four biologically independent samples examined over three independent experiments and expressed as mean ± SE; (NS) not significant. Statistical analysis was performed using an unpaired *t* test, Kolmogorov–Smirnov test. See experimental procedures for details of counting methods.

In our initial experiments, we incubated a whole neonatal mouse testis in 0.02% BC solution for 10 min and, after thorough washing, cultured the organ for 4 days at the surface/air interface in wells atop an agarose block (Fig. 1a). The testis was then fixed and stained for whole-mount immunofluorescence analysis using an antibody against the Sertoli cell marker SOX9 (*Sry-box 9*). In this assay, SOX9 + Sertoli cells were depleted throughout the testis (Fig. 1b, c). Neonatal testes incubated in PBS, 0.02% or 0.03% BC were sectioned, and Sertoli and other cell types were counted (Fig. 1; Supplementary Fig. 1a–l). A solution of 0.02% BC completely eliminated Sertoli cells in 82% of the tubule cross-sections analyzed (719/873 tubule sections, *n* = 4 independent testes), while fewer Sertoli cells were left in the remainder (952 cells/154 tubule sections; ~6 cells/tubule section; *n* = 4 independent testes) compared with control (3070 cells/115 tubule sections; ~27 cells/tubule section; *n* = 4 independent testes) (*P* = 0.029). Examples of most-to-least affected tubules are presented in Supplementary Fig. 1m–o.

We performed H&E staining on samples treated with 0.02% BC to confirm that Sertoli cells (and not only SOX9 protein) were lost. These assays showed that by day 3, there was a severe depletion of Sertoli cell nuclei along the basal lamina of seminiferous cords (Supplementary Fig. 2a, b). Apoptotic cell death increased from day 2 to day 4 based on staining with cleaved caspase 3 (Supplementary Fig. 2c, d). Loss of SOX9 + cells (Fig. 1b, c) was associated with elevated numbers of F4/80 + macrophages. However, despite the severe depletion of Sertoli cells based on both histology and SOX9 staining, the normal distribution of Laminin (LMN) showed that the structure of the seminiferous tubule was well maintained (Fig. 1d, e). Importantly, other cell types in the testis, 3βHSD (3β-hydroxysteroid dehydrogenase)-positive Leydig cells (Fig. 1f, g) were spared. Immunohistochemistry for smooth muscle actin, alpha (αSMA) suggested that PMCs were intact (Fig. 1h, i), and antibody staining with both the germ-cell-specific monoclonal antibody (TRA98)^16^ and GDNF family receptor alpha-1 (GFRα1) revealed that some germ cells remained along the basement membrane in Sertoli-ablated tubules (Fig. 1, j–m). Testes treated with 0.02% or 0.03% BC were sectioned, and the number of germ cells per tubule cross-section was counted. In samples treated with 0.02% BC, germ cell numbers were significantly reduced (~4 cells/tubule cross-section in a total of 968 cross-sections analyzed; *n* = 4 independent testes) compared with controls (~9 cells/tubules cross-section in a total of 340 tubules counted; *n* = 4 testes) (*P* = 0.029) (Fig. 1n), likely a secondary effect of Sertoli cell depletion. However, the numbers of PMCs and Leydig cells outside the tubules were not affected: 1303 PMCs/125 tubule cross-sections in treated testis (*n* = 4 independent testes) versus 963 cells/101 tubule cross-sections in control testis (*n* = 4 independent testes); and 3253 Leydig cells/873 tubules in treated testis (*n* = 4 independent testes) versus 1883 cells/541 tubules in control testis (*n* = 4 independent testes) (Fig. 1n). Although incubation of the neonatal testis with 0.03% BC led to a more severe loss of Sertoli cells, it also caused disruption of cord structure and loss of germ cells, PMCs, and Leydig cells (Supplementary Fig. 1c and Supplementary Fig. 2e, f).

### Time course of cell depletion in vivo

**Fig. 2:**
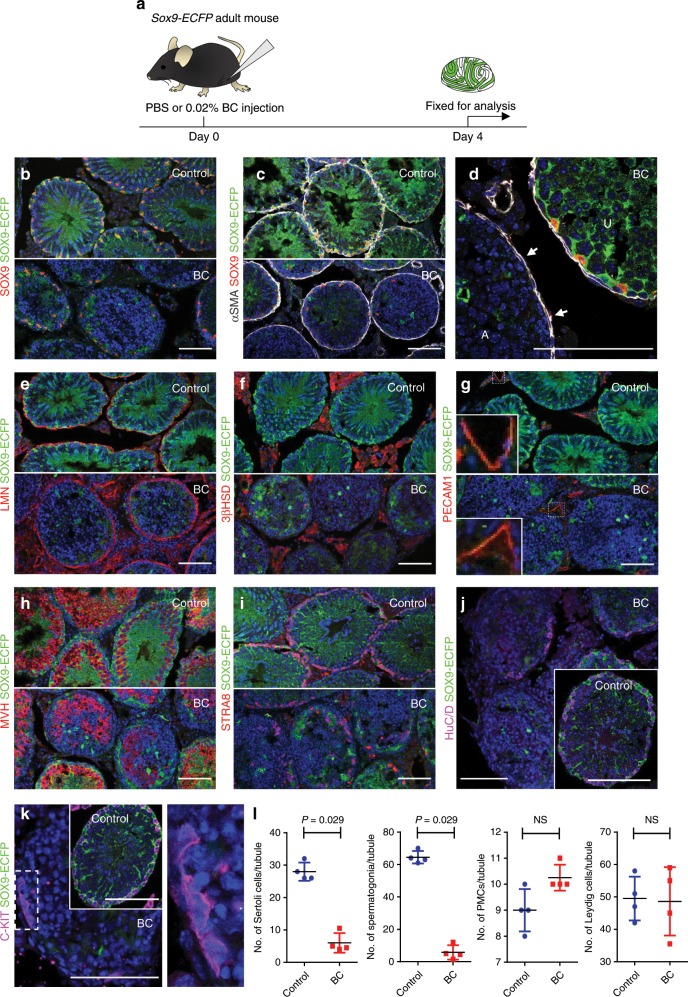
Four days after BC treatment in vivo, only Sertoli and germ cells were severely depleted. **a** Schematic representation of experimental procedures for in vivo adult Sertoli cell ablation. **b**–**k** *Sox9-ECFP* adult mouse testis 4 days after PBS or BC injection into seminiferous tubules. Tissues were stained with antibodies against ECFP (green; SOX9-ECFP, in this transgenic line, ECFP is present throughout the nucleus and cytoplasm of Sertoli cells) and Hoechst (blue). **b** Antibody staining of endogenous SOX9 (red); **c**, **d** αSMA (peritubular myoid cells; white; arrow). BC-affected tubule is marked “A”, and BC-unaffected tubule is marked “U”. **e** LMN-positive basement membrane (red). **f** Leydig cells (3βHSD-positive, red). **g** Vascular structures (PECAM1-positive, red) are shown. The left bottom corner of each frame (white box) shows a magnification of a vessel. **h** MVH-positive germ cells (red). **i** STRA8-positive spermatogonia (red). **j** HuC/D-positive spermatogonia (magenta) on the basement membrane in treated or untreated control (inset). **k** C-KIT-positive differentiated spermatogonia (magenta) in treated or untreated control (inset). The rectangular area surrounded by the broken line is enlarged on the right. Ten independent experiments. Scale bar: 100 µm. **l** Quantification of BC affect on Sertoli, germ cells, Leydig, and peritubular myoid cells. Data were analyzed from four biologically independent samples examined over three independent experiments and expressed as mean ± SD; (NS) not significant. Statistical analysis was performed using unpaired *t* test, Kolmogorov–Smirnov test.

To test the efficacy of BC in vivo, we injected a 0.02% BC solution or PBS into the rete testis of anesthetized adult male mice carrying the *Sox9-ECFP* transgene, which marks Sertoli cells (Fig. 2a). H&E staining and immunohistochemistry showed that many Sertoli cell nuclei disappear by day 4 (Supplementary Fig. 3a–d). This result was confirmed by loss of SOX9 + Sertoli cells from 27% of the tubule cross-sections analyzed (248/908, *n* = 4 independent testes) (Fig. 2b–l). In 6% of the tubule cross-sections (59/908, *n* = 4 testes), fewer Sertoli cells remained (an average of 6 Sertoli cells/tubule cross-section compared with an average of 28 Sertoli cells/tubule cross-section in testes injected with PBS) (*P* = 0.029). Analysis of affected testis tubules on day 3 showed defects in the differentiating germ cell layer, with many irregularly condensed nuclei (Supplementary Fig. 3e, f). Cleaved Caspase 3 + cells were rarely seen on day 3 (Supplementary Fig. 3e), but some cells, presumably spermatocytes, were positive for Caspase 3 on day 4 (Supplementary Fig. 3g). There was evidence for accumulation of F4/80 macrophages around the tubules, including invasion into the lumen of some tubules on day 4 (Supplementary Fig. 3h). These macrophages may have scavenged dying Sertoli cells and spermatocytes. In summary, on day 3, testes injected with BC showed the onset of cell degeneration within tubules. However, cell loss and inflammation were not observed until day 4.

Immunohistochemistry for αSMA suggested that PMC morphology was intact (Fig. 2c, d), and Laminin staining also showed an intact basal lamina surrounding affected tubules (Fig. 2e). Antibodies against 3βHSD and platelet/endothelial cell adhesion molecule 1 (PECAM1) revealed that Leydig cells and endothelial cells were not obviously affected (Fig. 2f, g). Although loss of Sertoli cells resulted in the rapid loss of differentiating germ cells (Fig. 2h), some surviving spermatogonia were present along the basal lamina in drug-affected tubules based on staining with antibodies against STRA8 (stimulated by retinoic acid gene) (Fig. 2i), HuC/D (human HuC/HuD neuronal protein) and C-KIT (Fig. 2j, k; Supplementary Fig. 4a, b). To quantify the effect of BC on other cell types in adult testis in vivo, the number of HuC/D + spermatogonia, Leydig cells, or PMCs per cross-section of BC-affected seminiferous tubules was counted (*n* = 4 independent testes). On day 4, the number of spermatogonia (positive for the spermatogonial marker, HuC/D) per tubule cross-section was decreased in drug-treated testes (*P* = 0.029). However, the numbers of Leydig cells and PMCs were similar to controls (Fig. 2l). In summary, 4 days after 0.02% BC treatment, Sertoli cells were significantly depleted, but the interstitial compartment, testis cord structure, and some spermatogonia were still present, and there was no evidence of an effect on the behavior or viability of the treated animals.

### Canine Sertoli cells are also depleted by drug treatment

To test whether BC can deplete Sertoli cells from the testis of other mammals, we used the same in vitro procedure on 6 to 8-week-old canine testis. Testis tissue fragments were incubated with PBS or 0.02% or 0.03% BC for 10 min, washed, and cultured for 3 days (Supplementary Fig. 5a, b). At the end of this time, testis fragments were fixed and stained for immunofluorescence analysis using antibodies against SOX9 and the germ cell marker MVH (mouse vasa homolog). In control tissue, both Sertoli cells and germ cells were detected in seminiferous tubules (Supplementary Fig. 5c). However, in 0.02% BC-treated testis tissue, Sertoli cells were depleted, particularly from the peripheral regions (Supplementary Fig. 5d). In 0.03% BC-treated tissues, all Sertoli cells were absent, but some spermatogonia survived in Sertoli-depleted seminiferous tubules (Supplementary Fig. 5e). Tubule structure was well maintained (Supplementary Fig. 5f, g). However, compared with controls (Supplementary Fig. 5f, h), the numbers of GATA4-positive Leydig cells were reduced in 0.03% BC-treated testes (Supplementary Fig. 5g), and αSMA-positive PMCs were almost completely lost (Supplementary Fig. 5i), similar to our observations in 0.03% BC-treated mouse testis (Supplementary Fig. 1c).

### Repopulation of Sertoli cells in BC-treated testis

**Fig. 3:**
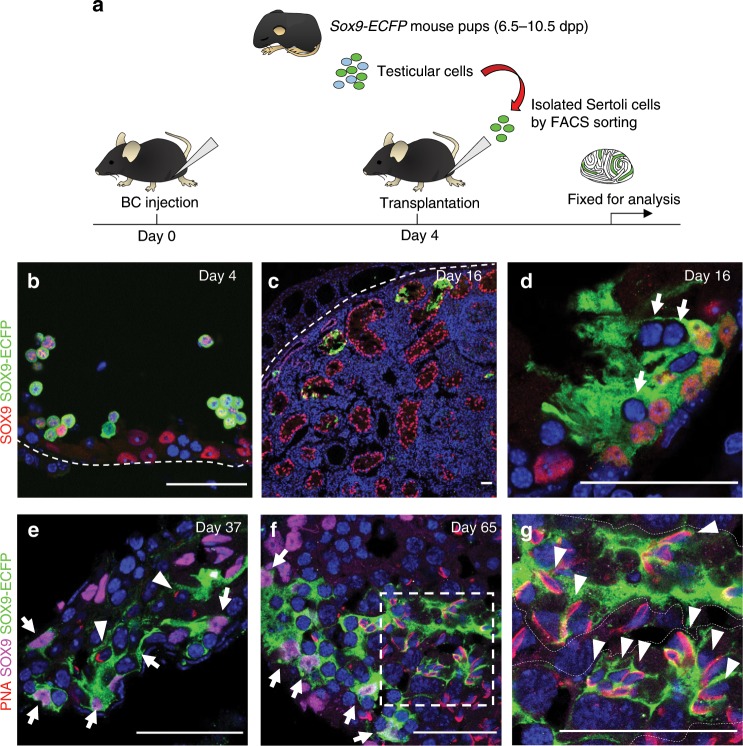
Transplantation of Sertoli cells reconstituted the germ cell niche and rescued spermatogenesis. **a** FACS-isolated Sertoli cells from 6.5–10.5 dpp pups were transplanted into adult testis 4 days after BC treatment. **b** On the day of transplantation (day 4), donor cells were detected in the lumen of seminiferous cords. Donor Sertoli cells were SOX9-ECFP (green, cytoplasmic stain), and also labeled with an antibody against native SOX9 (red, nuclear stain). Some host Sertoli cells remained, also labeled with native SOX9 (red), but not ECFP (green). **c**, **d** Twelve days after transplantation, donor Sertoli cells (SOX9-ECFP positive, green) isolated from 10.5 dpp pups colonized in some host seminiferous tubules and contacted host germ cells (arrows, cytoplasm surrounding round blue nucleus is not green). **e**–**g** Recipient testis stained with SOX9-ECFP (green), antibodies against SOX9 (magenta), the acrosomal marker PNA (red), and Hoechst (blue) are shown. Donor Sertoli cell contribution was observed in two in four biologically independent testis examined over two independent experiments. Arrows point to donor Sertoli cells with green cytoplasm. **e** Recipient testis 33 days after transplantation of Sertoli cells from 6.5 dpp *Sox9-ECFP* pups, showing that primitive spermatids (arrowhead), are present surrounded by donor Sertoli cells (green, arrow). **f** On day 65, donor Sertoli cells from 7.5 dpp *Sox9-ECFP* pups (green, arrows) supported spermatogenesis from host spermatogonia. Area within broken lines is enlarged in **g**, where elongated spermatids with a PNA-positive acrosomal cap were present (arrowheads) surrounded by donor Sertoli cell cytoplasm (green, within dotted lines). Scale bar: 50 µm.

To determine whether donor Sertoli cells can efficiently engraft in drug-depleted cords, we transplanted a purified population of Sertoli cells isolated by fluorescence-activated cell sorting (FACS) from an 6.5–10.5 days postpartum (dpp) *Sox9-ECFP* mouse testis (for analysis of this population, see Supplementary Fig. 6a, b) into an adult mouse testis prepared by injection of BC into the rete 4 days earlier (Fig. 3a). Soon after transplantation, some clusters of donor cells were found in the lumen (Fig. 3b). However, after 12 days, donor Sertoli cells colonized the basement membrane in some tubules (2–6/section) and surrounded host germ cells (Fig. 3c, d). Thirty-three days after transplantation, donor Sertoli cells were present near host spermatogonia that had differentiated to primitive spermatids based on PNA (peanut agglutinin) staining (Fig. 3e). Elongating spermatids were detected 8 weeks after transplantation (Fig. 3f, g) in two of four engrafted testes.

### Engraftment of multiple cell types into BC-treated testis

**Fig. 4:**
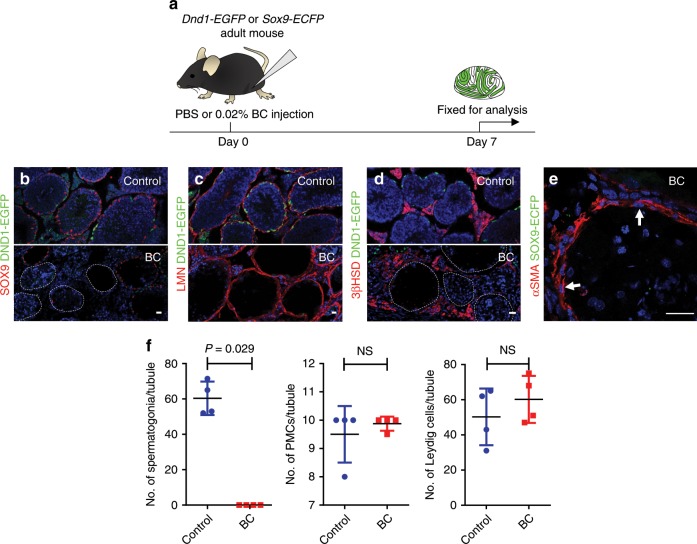
Spermatogonia were also lost by 7 days after BC treatment, but Leydig and PMCs were intact. **a** Schematic representation of experimental procedures for in vivo adult germ and Sertoli cell ablation. **b**–**d** DND1-EGFP (green; and see Supplementary Fig. 7) labels spermatogonia, co-labeled with antibodies against **b** SOX9 (red), **c** LMN (red), **d** 3βHSD (red). **e** SOX9-ECFP (green) and αSMA (red). Hoechst (blue). Ten independent experiments. Scale bar: 25 µm. **f** Quantification of spermatogonia, Leydig cells, and PMCs 7 days after 0.02% BC treatment. Data were analyzed from four biologically independent samples examined over three independent experiments and expressed as mean ± SD; (NS) not significant. Statistical analysis was performed using an unpaired *t* test, Kolmogorov–Smirnov test.

We used a *DND1 (dead end homolog 1)-EGFP* mouse to investigate spermatogonia kinetics after BC treatment. In this transgenic line, all spermatogonia, including undifferentiated spermatogonia and differentiating spermatogonia express EGFP (Supplementary Fig. 7, a–d). Seven days after BC injection, in 24% of tubule cross-sections (205/868; *n* = 4 independent testes), both Sertoli cells and germ cells, including spermatogonia, had disappeared (Fig. 4a, b). Although Sertoli cells and germ cells/spermatogonia were lost 7 days after treatment, cord structure, Leydig cells, and PMCs were intact (Fig. 4, c–e), and their numbers surrounding BC-affected seminiferous tubules were similar to controls (*n* = 4 independent testes) (Fig. 4f).

**Fig. 5:**
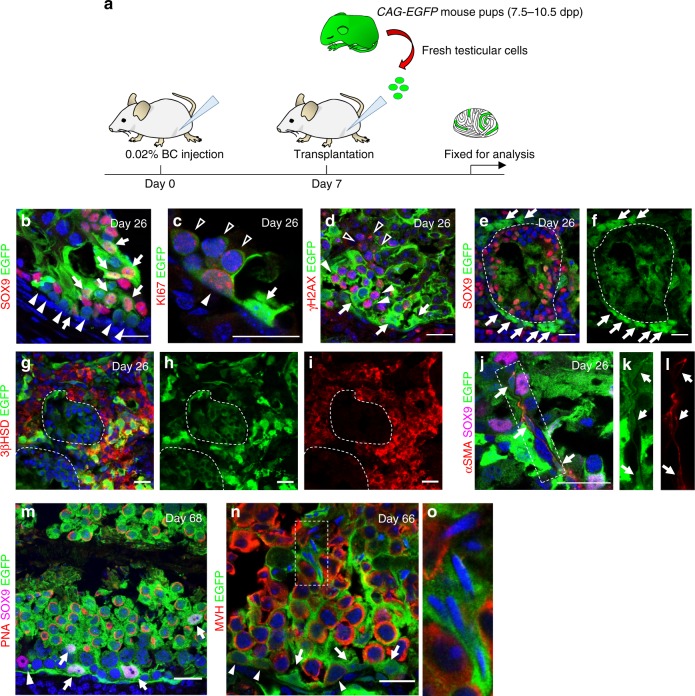
Fresh donor testicular cells injected 7 days post-BC treatment reconstitute spermatogonial niche. **a** Schematic representation of experimental procedures for testicular cell transplantation 7 days after BC treatment. **b**–**g** Host testis 19 days after testicular cell transplantation from 9 dpp *CAG-EGFP* pups. CAG-EGFP (green) labels all transplanted cells. **b** Donor Sertoli cells (arrow, green) with nuclear SOX9 (red) and donor spermatogonia (arrowhead, round cells with large nuclei surrounded by green halo) colonize within cords. **c** Spermatocytes generated from donor spermatogonia (open arrowheads). Donor Sertoli cell (arrow) and donor spermatogonia (white arrowhead) positive for active cell cycle marker, KI67 (red). **d** Donor Sertoli cell (arrow) fosters donor spermatocytes: leptotene spermatocytes (white arrowhead), pachytene spermatocytes (open arrowhead) label with γH2AX (red). **e**, **f** In addition to donor cells inside tubules, some of which express SOX9 (red), many SOX9-negative cells colonized outside of tubules. **g**–**i** Donor Leydig cells expressing 3βHSD (red) colonized the interstitium, and **j**–**l** donor PMCs (arrow; αSMA (red)) surrounded testis cords (region within dotted rectangle is magnified to the right in the green channel; green myoid cell is outlined in red). SOX9 (magenta). **m** Recipient testis 61 days after 7 dpp *CAG-EGFP* testicular cell transplantation. SOX9 (magenta) labels both donor Sertoli cells (arrows) and a remaining host Sertoli cell (arrowhead). PNA (red) co-labels many donor spermatogonia (green). **n** Host testis 59 days after testicular cell transplantation from 10 dpp *CAG-EGFP* donors. Donor Sertoli cells (arrow, green) surround elongating sperm labeled with MVH (red). Donor-derived spermatogonia persist on the basement membrane (arrowhead). **o** Higher magnification view of the region surrounded by broken lines shows elongating sperm. Donor spermatogenesis with multiple cell type colonization was observed in 9 of 13 biologically independent testes examined over four independent experiments. Scale bar: 25 µm.

To determine whether the host testes depleted for both Sertoli and germ cells could be used as a scaffold for engraftment of multiple cell types, we performed testicular cell transplantation 7 days post-BC treatment (Fig. 5a). Nineteen days after transplantation into a BC-treated host testis, donor Sertoli cells and spermatogonia had colonized in ~4–8% of host tubules (Fig. 5b). A highly colonized region of a testis isolated 59 days posttransplantation is shown in Supplementary Fig. 8a, b. Donor spermatogonia proliferated with support from donor Sertoli cells (Fig. 5c), initiated meiosis, and progressed to the spermatocyte stage. EGFP-positive leptotene and pachytene spermatocytes were detected (Fig. 5d). Other donor testicular cells, negative for SOX9, were observed around some tubules where donor Sertoli cells and spermatogonia colonized (Fig. 5e–l). Most of these interstitial EGFP-bright donor cells were Leydig cells (Fig. 5g–i), but some donor PMC colonization was also detected (Fig. 5j–l). Because these cells were observed specifically around the seminiferous tubules where donor Sertoli cells and germ cells colonized, we speculated that they migrated into the interstitium from the drug-affected tubules. Eight weeks after transplantation, PNA-positive spermatids were observed in tubules colonized by donor Sertoli cells and spermatogonia (Fig. 5m). Round spermatids and elongated spermatids generated from donor spermatogonia with support from donor Sertoli cells were detected in the host testis (Fig. 5m–o). In 9/13 testes, donor Sertoli cell and spermatogonia colonization was observed, and advanced spermatogenesis was detected in four of these hosts.

### Transplantation of cryopreserved testicular cells

**Fig. 6:**
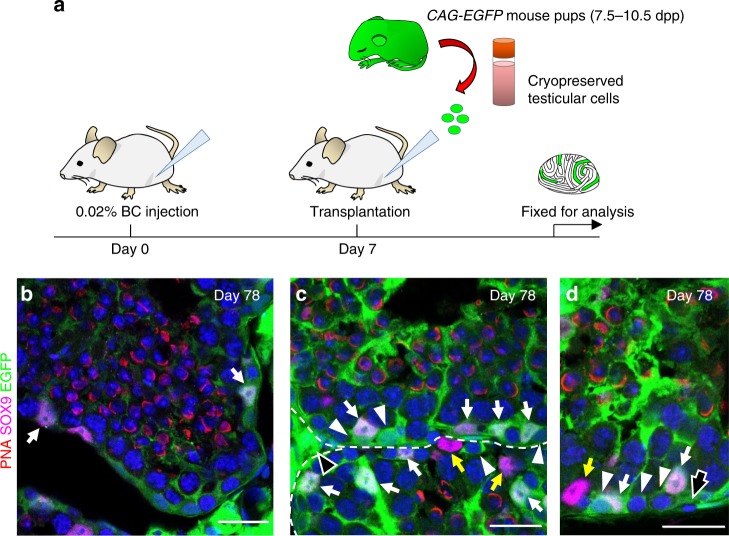
Cryopreserved donor testicular cells injected 7 days post-BC treatment reconstitute multiple cell types in the testis. **a** Schematic representation of experimental procedures for cryopreserved testicular cell transplantation 7 days after BC treatment. **b**–**d** Host testis 71 days after transplantation of 9 dpp *CAG-EGFP* testicular cells that had been cryopreserved for 39 days. CAG-EGFP (green), PNA (red), SOX9 (magenta), and Hoechst (blue). **b** Cryopreserved donor Sertoli cells (green), co-labeled with antibodies against SOX9 (magenta; arrows), colonized and supported host spermatogenesis (PNA (red) marks round spermatids that are not green). **c**, **d** Cryopreserved donor Sertoli cells (green, with magenta nuclei, white arrows) colonized beside donor spermatogonia (white arrowheads) and supported donor spermatogenesis (PNA (red) marks round spermatids from the donor (green)). Host Sertoli cells (yellow arrows) were also present. **c** Donor Leydig cells (black arrowhead) and **d** donor peritubular myoid cell (black arrow) were also observed. Hoechst (blue). Cryopreserved donor advanced spermatogenesis with supporting cell colonization was observed in 2/3 biologically independent testis in two independent experiments. Scale bar: 25 µm.

To test the efficiency of engraftment using cryopreserved donor tissue, we dissociated testis tissue from *CAG-EGFP* pups and cryopreserved the cells for 39 days. Seven days after BC treatment of wild-type host mice, cryopreserved donor testicular cells were thawed and introduced into the rete testis. The results were similar to the results using freshly prepared cells for transplantation. Seventy-one days after transplantation, we found that cryopreserved Sertoli cells and spermatogonia had colonized testis tubules and donor supporting cells, presumably Leydig cells and PMCs, were found in the interstitium (Fig. 6, a–d; Supplementary Fig. 8c, d). In some tubules, cryopreserved Sertoli cells supported host spermatogenesis (Fig. 6b), and in other tubules, cryopreserved donor Sertoli cells colonized with donor spermatogonia and supported donor spermatogenesis (Fig. 6c, d). In 3/3 host testes, cryopreserved Sertoli cells and spermatogonia colonized the tubules, and advanced spermatogenesis from donor cells was observed in two of these hosts.

## Discussion

Defects in Sertoli cells and other somatic cell types that form the spermatogonial niche in the testis are responsible for many cases of infertility^2–4^, but efforts to replace defective Sertoli cells in vivo have been hampered by the inability to achieve engraftment in competition with resident cells^14^. Ablation of Sertoli cells was previously reported using diphtheria toxin receptor-mediated conditional and targeted cell ablation systems^14,17,18^. However, while these methods have significantly advanced our knowledge of testis biology, they are not feasible in the clinic.

We have devised a technique to deplete Sertoli cells using a nontoxic agent, benzalkonium chloride. BC was previously reported to remove mouse and rat enteric nerve plexi when the GI tract was treated in vitro^19–25^. In this study, 0.02% BC injection into the rete testis caused no discernable side effects in the mouse, but did cause severe Sertoli cell depletion and partial depletion of spermatogonia and differentiating sperm. BC is a cationic surfactant that works preferentially on cells with a strong negative membrane charge^26^, raising the possibility that the Sertoli cell membrane might have a more negative charge than other cell types in the testis. Future experiments will be designed to address the mechanism of BC action. We hypothesize that the most depleted tubules were those most closely connected to the rete site of injection. We show that the effect of incubation with BC is similar in canine testes, which suggests that the treatment can be used in large mammals. In all, 0.03% BC treatment of mouse and canine testes in vitro led to Sertoli cell elimination, but also caused PMC depletion and reduced the number of Leydig and germ cells. These results indicate that the dose and time to engraftment will require optimization for each species.

Alongside this discovery, we developed methods for engraftment of Sertoli cells or multiple testis cell types. An ultrasound-guided seminiferous tubule transplantation technique has already been established for primates and humans^27^, thus our method is technically feasible for clinical applications. Sertoli cells used for engraftment could be derived via an induced pluripotent stem cell (iPSC) strategy^28^ to avoid immune rejection. In cases where the defect in Sertoli cells is identified through whole-exon sequencing and cross-referencing with known Sertoli essential genes (eg. *KITL*), it could be corrected via CRISPR-mediated genome editing strategies in iPSCs prior to transplantation following BC depletion. A proof-of-principle experiment demonstrating this approach in mice would be an important follow-up experiment. Testing the fertility of sperm generated with support from donor Sertoli cells is not feasible in this experiment, because we cannot distinguish host sperm matured in the company of donor Sertoli cells from sperm matured in regions of the host testis where host Sertoli cells survived BC treatment. In future experiments, we will replace Sertoli cells in the *Kitl*^*Sl/Sld*^ mutant mouse, which lacks spermatogenesis due to a Sertoli cell defect, and perform micro insemination to test fertility.

Since seminiferous transplantation was developed by Brinster^29^, xenogeneic transplantation has been investigated. Although human and piglet spermatogonia attach to the mouse testis basement membrane and remain for several months, they do not show further maturation^30–32^, suggesting that the mouse germ cell niche cannot support xenogeneic spermatogonia. Here, we show that cryopreserved Sertoli cells, Leydig cells, and PMCs can colonize the BC-treated testis, regenerate the germ cell niche, and support spermatogenesis. Replacement of many cell types comprising the germ cell niche might make it possible to produce human sperm in an immunocompromised BC-treated mouse testis from cryopreserved tissue of pre-pubertal donors.

In summary, we have identified an FDA approved, safe drug that induces severe Sertoli cell depletion when added to isolated mouse or canine testes in vitro or injected into the rete testis of mice in vivo. Using this drug, we have developed techniques for Sertoli-only transplantation and for transplantation of spermatogonia plus multiple supporting cell types. We hope to use these approaches to analyze the function of Sertoli cells, design a therapy for human idiopathic male infertility, and investigate the cross talk between niche cells and stem cells. Finally, if xenogeneic spermatogenesis can be achieved, it may not only be valuable for treatment of infertility for young cancer patients but may also be adapted to produce sperm from humans or endangered species in mice.

## Methods

### Animals

CD1 outbred, C57BL/6J and FVB inbred strains (from The Jackson Laboratory), and FVB *CAG-EGFP*^33^, C57BL/6J *Sox9-ECFP*^34^ and hybrid (B6SJLF1/J x CD1) *Dnd1-EGFP* (Ruthig et al. in preparation) transgenic mice were used. For Sertoli cell transplantation experiments, C57BL/6J *Sox9-ECFP* pups were used as donors on 6.5–10.5 dpp into adult C57BL/6J or F1 (FVB x C57BL/6J) hosts. FVB *CAG-EGFP* mouse pups were used as donors on 7.5–10.5 dpp into adult FVB or F1(C57BL/6J x FVB) hosts. Mice were maintained in a barrier facility, using an individually ventilated cage system (Allentown; PNC75JU160SPCD3). The light cycle was maintained as 12 h on and 12 h off. The colony was maintained at a temperature of 22  ± 0.1 °C, with humidity at 30–70%. Experimental protocols were approved by the Institutional Animal Care and Use Committee of Duke University Medical Center. Castrated 6- and 8-week-old dog testes were provided from the Animal Protection Society of Durham, NC.

### BC solution preparation

A commercially available solution (20% w/v) of benzalkonium chloride was obtained (Acros Organics; CAS;8001–54–5) and diluted with PBS to prepare 0.02% and 0.03% solutions.

### Testis tissue culture method

In total, 0.5 or 1.5 dpp neonatal whole testes were dissected from CD1, incubated with 0%, 0.02% or 0.03% BC for 10 min, washed several times with PBS, and placed atop 1.5% agarose blocks (Difco; 214530) in six-well tissue culture plates (Falcon; 353046) with ~500 μl of DMEM (Gibco; 11995–065), supplemented with 10% FBS (Gibco; 10438–026), and penicillin and streptomycin (P/S), to reach a level halfway up the side of the block. After culture, tissues were washed in PBS and fixed in 4% PFA (wt/vol) at 4 °C overnight for immunofluorescence. Fragments (2 × 2 × 2 mm) of the canine testis tissues were incubated with 0%, 0.02%, or 0.03% BC for 10 min and cultured atop agar blocks in DMEM + P/S with the addition of 10% KnockOut Serum Replacement (KSR) (Invitrogen; 10828–028) replacing FBS^35^. During all incubations, the culture incubator was supplied with 5% (vol/vol) carbon dioxide in air and maintained at 37 °C.

### Drug injection into seminiferous tubules

Host mice were anesthetized using Avertin^36^. A glass needle (World Precision Instruments; TW100–4) was pulled from a vertical pipette puller (World Precision Instruments; PLU-100) (setting; Heat 515, Pull 100, Trip 75, delay 75) and back-loaded with 20 μl of injection mixture, containing the drug and 0.02% bromophenol blue (Sigma; 114391) to track the success of the injection and extent of delivery into the tissue. The testis was removed from the scrotum and held gently by forceps. The back of the glass needle was connected to a polyethylene tube linked to a mouth pipette. The distal portion of the efferent duct bundle was held with forceps, and the tip of the glass needle was inserted into the bundle and pushed along the efferent ducts toward the testis, stopping when the tip reached the junction between the efferent ducts and the testis, where 10–15 μl of the solution was gently injected.

### Donor Sertoli cell preparation

On the day of transplantation, FACS analysis was used to isolate donor Sertoli cells expressing SOX9-ECFP^37^. Testes of *Sox9-ECFP* mouse pups were dissected. Testicular cells were enzymatically dissociated using 2 mg/ml collagenase type IV (Gibco; 17104019) for 15 min followed by incubation in 0.25% trypsin and 1 mM EDTA for 10 min at 37 ^o^C. The reaction was stopped by addition of serum, and cells were washed twice with DMEM supplemented with 10% FBS and P/S. The cell suspension was filtered through a 40-μm nylon mesh and resuspended in 10% FBS + DMEM + P/S. FACS was performed by the Duke Comprehensive Cancer Center Flow Cytometry Shared Resource. The positive fraction was pelleted, the liquid supernatant was removed, and the cells were resuspended in 10% FBS + DMEM + P/S.

### Cryopreservation and thawing method

After dissociation and washing steps, samples were placed in 2.0 ml cryovials (Corning; CLS430488) containing 500 μl of cryopreservation medium, (10% FBS + DMEM + P/S containing 10% DMSO). Samples were placed in a controlled freezing device (Mr. Frosty™ Freezing Container, Thermo Fisher Scientific) and cooled in a −80 °C freezer overnight. The following morning the cryovials were transferred to liquid nitrogen for storage. Prior to use, samples were thawed quickly in a bath warmed to 37 °C.

### Sertoli and whole testicular cell transplantation

Cell viability counts were performed +/− cryopreservation. The survival rate of cells in the suspension was determined using trypan blue (Thermo Scientific; 15250–061) and found to be high (80–90%). The solution was diluted with 10% FBS + DMEM + P/S to a concentration of 1 × 10^6^/ml isolated Sertoli cells or 1 × 10^8^/ml testicular cells, and 10–15 μl were injected through the rete testis into the seminiferous tubules of a host mouse on day 4 or day 7 after BC treatment. A glass needle (pulled as for drug injection) was back-loaded with the cell suspension and 0.04% Trypan blue to track the efficiency of the injection.

### Whole-mount immunofluorescent cytochemistry

For cultured neonatal mouse testes, whole-mount immunofluorescence analysis was performed. Samples were fixed overnight with 4% (wt/vol) paraformaldehyde in PBS at 4 °C. After several washes in PBS, samples were processed through a methanol gradient (25%, 50%, 75% and 100% in PBS) and stored in 100% methanol at −20 °C until use. Samples were re-hydrated and washed in PBTx (PBS, 1% Triton X-100), samples were incubated in blocking solution (PBTx, 10% FBS and 3% BSA) for 1 h at room temperature. Primary antibodies were diluted in blocking solution and applied to samples for 1 to 2 days at 4 °C. After several washes in 1% PBTx, fluorophore-conjugated secondary antibodies were applied for 2 days at 4 °C as described^38^. After several washes in PBS, samples were mounted in polyvinyl alcohol (MP Biomedicals; 151937) and imaged with a Zeiss 780 upright confocal laser microscope.

### Immunofluorescent cytochemistry on cryosections

Neonatal and adult mouse testis and canine testis tissues were fixed in 4% paraformaldehyde in PBS at 4 °C overnight, processed through a sucrose gradient (10%, 15%, and 20% in PBS), and cryo-embedded in OCT compound (Sakura Finetek)^38^. Samples were cut into 8–12-μm cryosections. Samples were incubated with blocking solution (0.1% PBTx + 10% FBS + 3% BSA) for 30 min, and primary antibodies were diluted in blocking solution and incubated overnight at 4 °C. Samples were washed several times in 0.1% PBTx and incubated with fluorophore-conjugated secondary antibodies for 1 h at room temperature. Samples were mounted after several washes in PBS. Primary and secondary antibodies used for immunofluorescence are listed in Supplementary Tables 1 and 2. Nuclei were counterstained with Hoechst 33342 dye. Samples were mounted in polyvinyl alcohol and imaged with a Zeiss 780 upright confocal laser scanning microscope.

### Immunofluorescent cytochemistry on FACS-isolated cells

Testicular cells were cultured on glass coverslips for 30 min at room temperature and fixed in 4% paraformaldehyde in PBS at room temperature for 20 min. Cells were incubated with blocking solution (0.1% PBTx + 10% FBS + 3% BSA) for 20 min, and primary antibodies against GFP and MVH were diluted in blocking solution and incubated at room temperature for 20 min. Samples were washed several times in 0.1% PBTx and incubated with fluorophore-conjugated secondary antibodies for 20 min at room temperature. Samples were mounted in polyvinyl alcohol after several washes in PBS and imaged with a Zeiss 780 upright confocal laser scanning microscope.

### Quantitative analysis using immunohistochemical sections

The Fiji version 1.52 g was used to count the proportion of tubules affected by BC^39^. This proportion was estimated by counting the total number of seminiferous tubule cross-sections (defined by αSMA staining) in 10–24 serial sections, and determining the proportion of tubule cross-sections not containing Sertoli cells (SOX9 + cells). The number of Sertoli cells in tubules, and the numbers of germ cells (TRA98 + cells in neonatal testis and HuC/D + spermatogonia in adult samples) were determined. Leydig cells (3βHSD + cells) and PMC (αSMA + cells) were counted in the interstitial areas immediately surrounding each cord. The GraphPad Prism version 6 was used for statistical analysis. Two-thirds of the values fall within the top and bottom bars. Statistical significance was determined by unpaired *t* tests, Kolmogorov–Smirnov test.

### Reporting summary

Further information on research design is available in the Nature Research Reporting Summary linked to this article.

## Supplementary information

Supplementary Information

Reporting Summary

## Data Availability

All relevant data are available from the corresponding authors on request.
