# Supplementary material for: Sertoli cell ablation and replacement of the spermatogonial niche in mouse
Source: Nat Commun. 2020 Jan 2;11:40. doi: 10.1038/s41467-019-13879-8 (PMC6940386; doi:10.1038/s41467-019-13879-8)
Supplement: Supplementary file 1 — Supplementary Information [file 41467_2019_13879_MOESM1_ESM.docx]

Supplemental Information for

**Sertoli cell ablation and replacement of the spermatogonial niche in mouse**

Tetsuhiro Yokonishi, Jennifer McKey, Shintaro Ide and Blanche Capel

**Content**

-Supplemental Figures 1-8

-Supplemental Tables 1-2


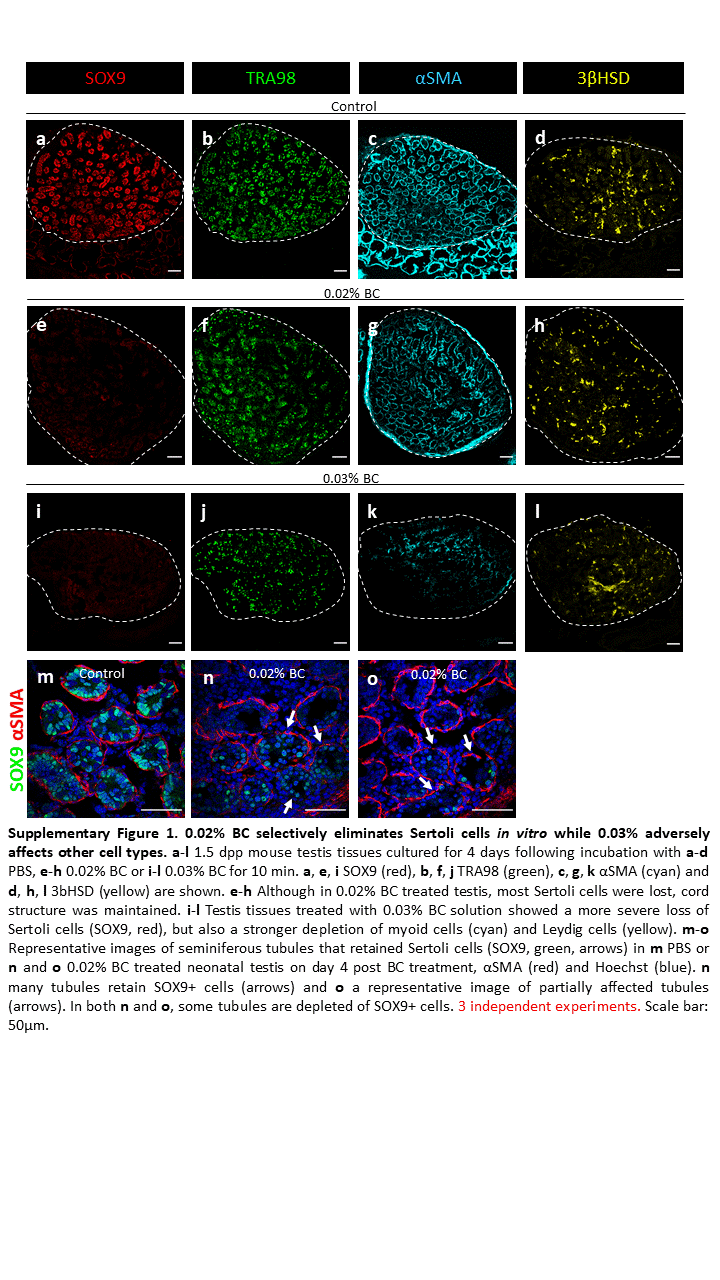


**Supplementary Figure 1. 0.02% BC selectively eliminates Sertoli cells *in vitro* while 0.03% adversely affects other cell types.** **a**-**l** 1.5 dpp mouse testis tissues cultured for 4 days following incubation with **a**-**d** PBS, **e**-**h** 0.02% BC or **i**-**l** 0.03% BC for 10 min. **a**, **e**, **i** SOX9 (red), **b**, **f**, **j** TRA98 (green), **c**, **g**, **k** αSMA (cyan) and **d**, **h**, **l** 3bHSD (yellow) are shown. **e**-**h** Although in 0.02% BC treated testis, most Sertoli cells were lost, cord structure was maintained. **i**-**l** Testis tissues treated with 0.03% BC solution showed a more severe loss of Sertoli cells (SOX9, red), but also a stronger depletion of myoid cells (cyan) and Leydig cells (yellow). **m**-**o** Representative images of seminiferous tubules that retained Sertoli cells (SOX9, green, arrows) in **m** PBS or **n** and **o** 0.02% BC treated neonatal testis on day 4 post BC treatment, αSMA (red) and Hoechst (blue). **n** many tubules retain SOX9+ cells (arrows) and **o** a representative image of partially affected tubules (arrows). In both **n** and **o**, some tubules are depleted of SOX9+ cells. 3 independent experiments. Scale bar: 50µm.


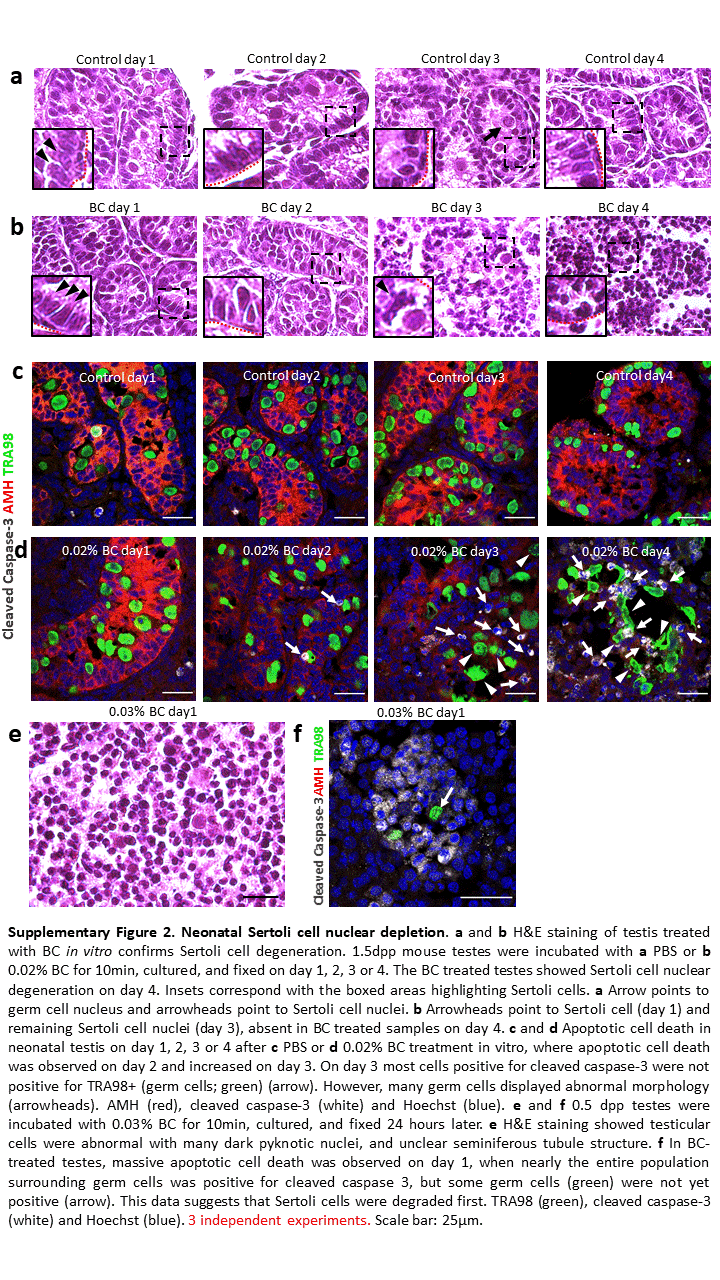


**Supplementary Figure 2. Neonatal Sertoli cell nuclear depletion**. **a** and **b** H&E staining of testis treated with BC *in vitro* confirms Sertoli cell degeneration. 1.5dpp mouse testes were incubated with **a** PBS or **b** 0.02% BC for 10min, cultured, and fixed on day 1, 2, 3 or 4. The BC treated testes showed Sertoli cell nuclear degeneration on day 4. Insets correspond with the boxed areas highlighting Sertoli cells. **a** Arrow points to germ cell nucleus and arrowheads point to Sertoli cell nuclei. **b** Arrowheads point to Sertoli cell (day 1) and remaining Sertoli cell nuclei (day 3), absent in BC treated samples on day 4. **c** and **d** Apoptotic cell death in neonatal testis on day 1, 2, 3 or 4 after **c** PBS or **d** 0.02% BC treatment in vitro, where apoptotic cell death was observed on day 2 and increased on day 3. On day 3 most cells positive for cleaved caspase-3 were not positive for TRA98+ (germ cells; green) (arrow). However, many germ cells displayed abnormal morphology (arrowheads). AMH (red), cleaved caspase-3 (white) and Hoechst (blue). **e** and **f** 0.5 dpp testes were incubated with 0.03% BC for 10min, cultured, and fixed 24 hours later. **e** H&E staining showed testicular cells were abnormal with many dark pyknotic nuclei, and unclear seminiferous tubule structure. **f** In BC-treated testes, massive apoptotic cell death was observed on day 1, when nearly the entire population surrounding germ cells was positive for cleaved caspase 3, but some germ cells (green) were not yet positive (arrow). This data suggests that Sertoli cells were degraded first. TRA98 (green), cleaved caspase-3 (white) and Hoechst (blue). 3 independent experiments. Scale bar: 25µm.


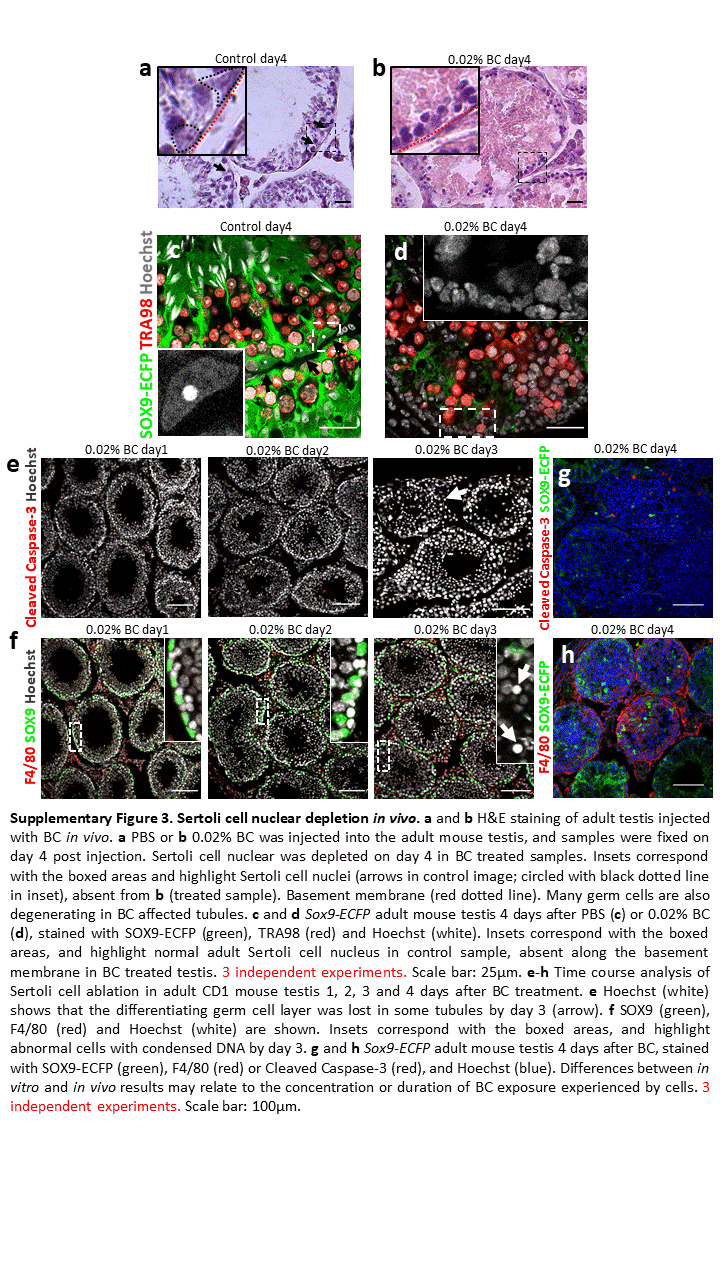


**Supplementary Figure 3. Sertoli cell nuclear depletion *in vivo*. a** and **b** H&E staining of adult testis injected with BC *in vivo*. **a** PBS or **b** 0.02% BC was injected into the adult mouse testis, and samples were fixed on day 4 post injection. Sertoli cell nuclear was depleted on day 4 in BC treated samples. Insets correspond with the boxed areas and highlight Sertoli cell nuclei (arrows in control image; circled with black dotted line in inset), absent from **b** (treated sample). Basement membrane (red dotted line). Many germ cells are also degenerating in BC affected tubules. **c** and **d** *Sox9-ECFP* adult mouse testis 4 days after PBS (**c**) or 0.02% BC (**d**), stained with SOX9-ECFP (green), TRA98 (red) and Hoechst (white). Insets correspond with the boxed areas, and highlight normal adult Sertoli cell nucleus in control sample, absent along the basement membrane in BC treated testis. 3 independent experiments. Scale bar: 25µm. **e**-**h** Time course analysis of Sertoli cell ablation in adult CD1 mouse testis 1, 2, 3 and 4 days after BC treatment. **e** Hoechst (white) shows that the differentiating germ cell layer was lost in some tubules by day 3 (arrow). **f** SOX9 (green), F4/80 (red) and Hoechst (white) are shown. Insets correspond with the boxed areas, and highlight abnormal cells with condensed DNA by day 3. **g** and **h** *Sox9-ECFP* adult mouse testis 4 days after BC, stained with SOX9-ECFP (green), F4/80 (red) or Cleaved Caspase-3 (red), and Hoechst (blue). Differences between *in vitro* and *in vivo* results may relate to the concentration or duration of BC exposure experienced by cells. 3 independent experiments. Scale bar: 100µm.


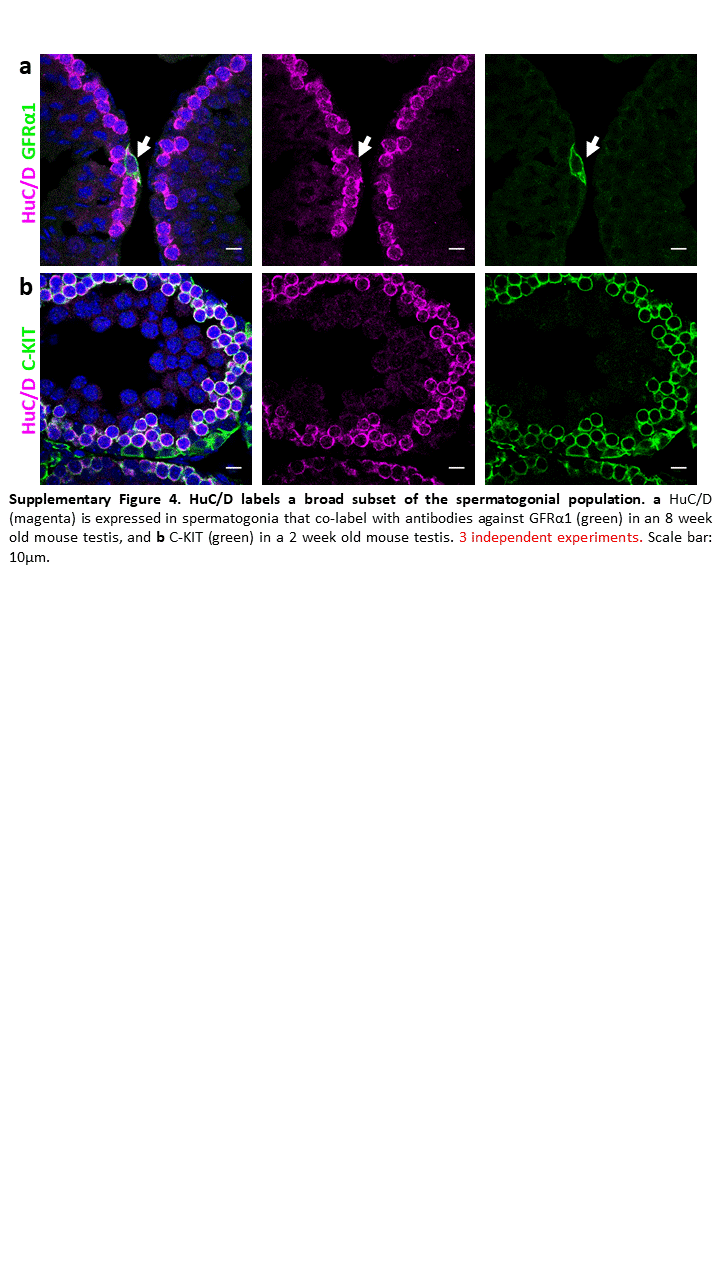


**Supplementary Figure 4. HuC/D labels a broad subset of the spermatogonial population. a** HuC/D (magenta) is expressed in spermatogonia that co-label with antibodies against GFRα1 (green) in an 8 week old mouse testis, and **b** C-KIT (green) in a 2 week old mouse testis. 3 independent experiments. Scale bar: 10µm.


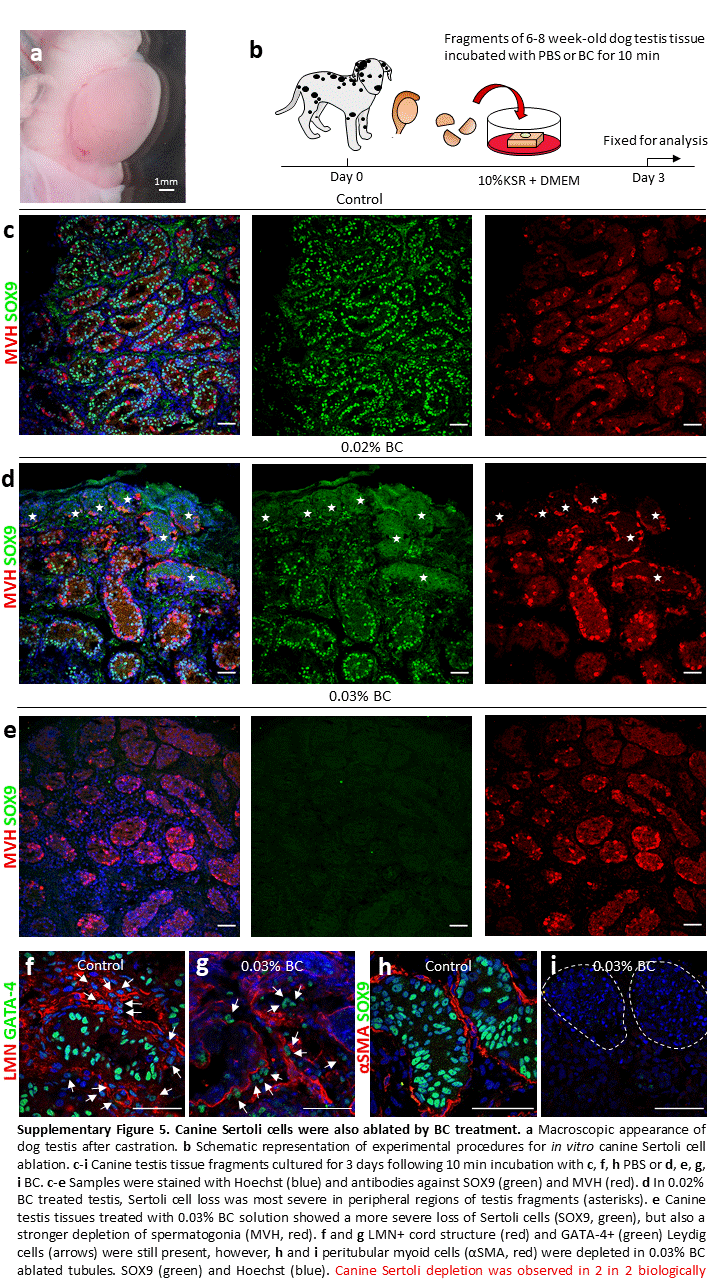


**Supplementary Figure 5. Canine Sertoli cells were also ablated by BC treatment.** **a** Macroscopic appearance of dog testis after castration. **b** Schematic representation of experimental procedures for *in vitro* canine Sertoli cell ablation. **c**-**i** Canine testis tissue fragments cultured for 3 days following 10 min incubation with **c**, **f**, **h** PBS or **d**, **e**, **g**, **i** BC. **c**-**e** Samples were stained with Hoechst (blue) and antibodies against SOX9 (green) and MVH (red). **d** In 0.02% BC treated testis, Sertoli cell loss was most severe in peripheral regions of testis fragments (asterisks). **e** Canine testis tissues treated with 0.03% BC solution showed a more severe loss of Sertoli cells (SOX9, green), but also a stronger depletion of spermatogonia (MVH, red). **f** and **g** LMN+ cord structure (red) and GATA-4+ (green) Leydig cells (arrows) were still present, however, **h** and **i** peritubular myoid cells (αSMA, red) were depleted in 0.03% BC ablated tubules. SOX9 (green) and Hoechst (blue). Canine Sertoli depletion was observed in 2 in 2 biologically independent testis examined over 2 independent experiments. Scale bar: 50µm.


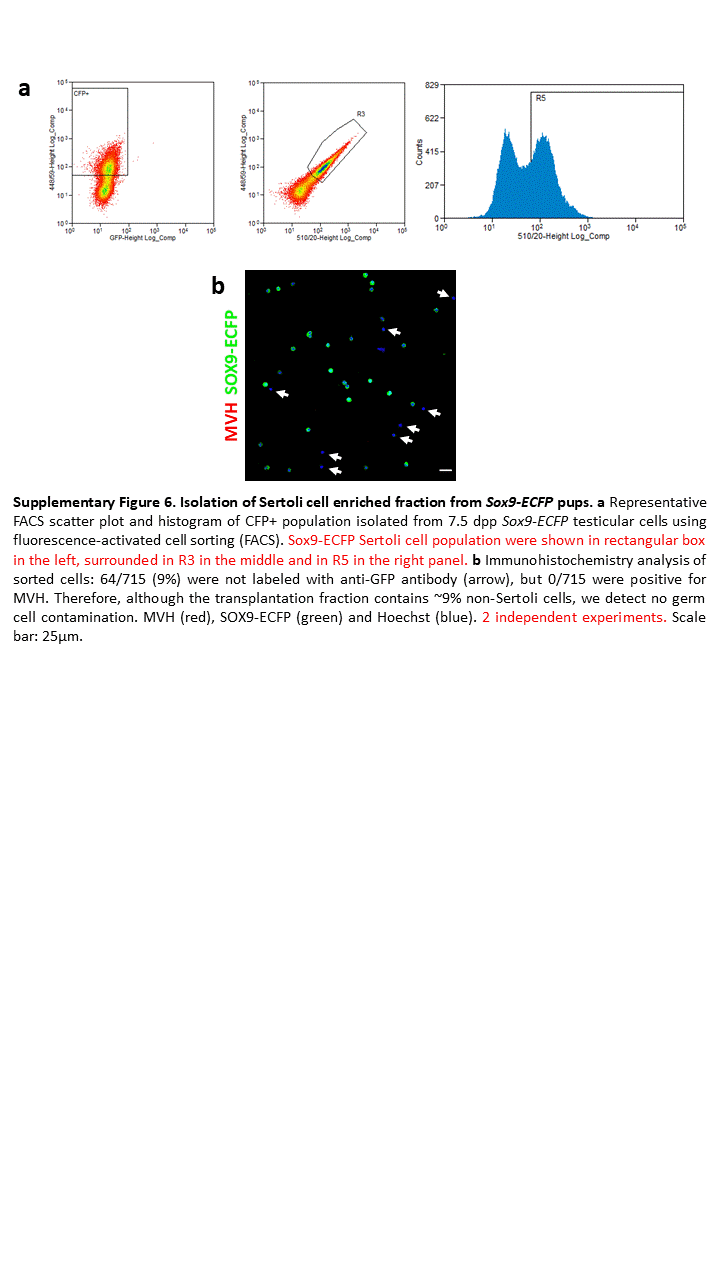


**Supplementary Figure 6. Isolation of Sertoli cell enriched fraction from *Sox9-ECFP* pups.** **a** Representative FACS scatter plot and histogram of CFP+ population isolated from 7.5 dpp *Sox9-ECFP* testicular cells using fluorescence-activated cell sorting (FACS). Sox9-ECFP Sertoli cell population were shown in rectangular box in the left, surrounded in R3 in the middle and in R5 in the right panel. **b** Immunohistochemistry analysis of sorted cells: 64/715 (9%) were not labeled with anti-GFP antibody (arrow), but 0/715 were positive for MVH. Therefore, although the transplantation fraction contains ~9% non-Sertoli cells, we detect no germ cell contamination. MVH (red), SOX9-ECFP (green) and Hoechst (blue). 2 independent experiments. Scale bar: 25µm.


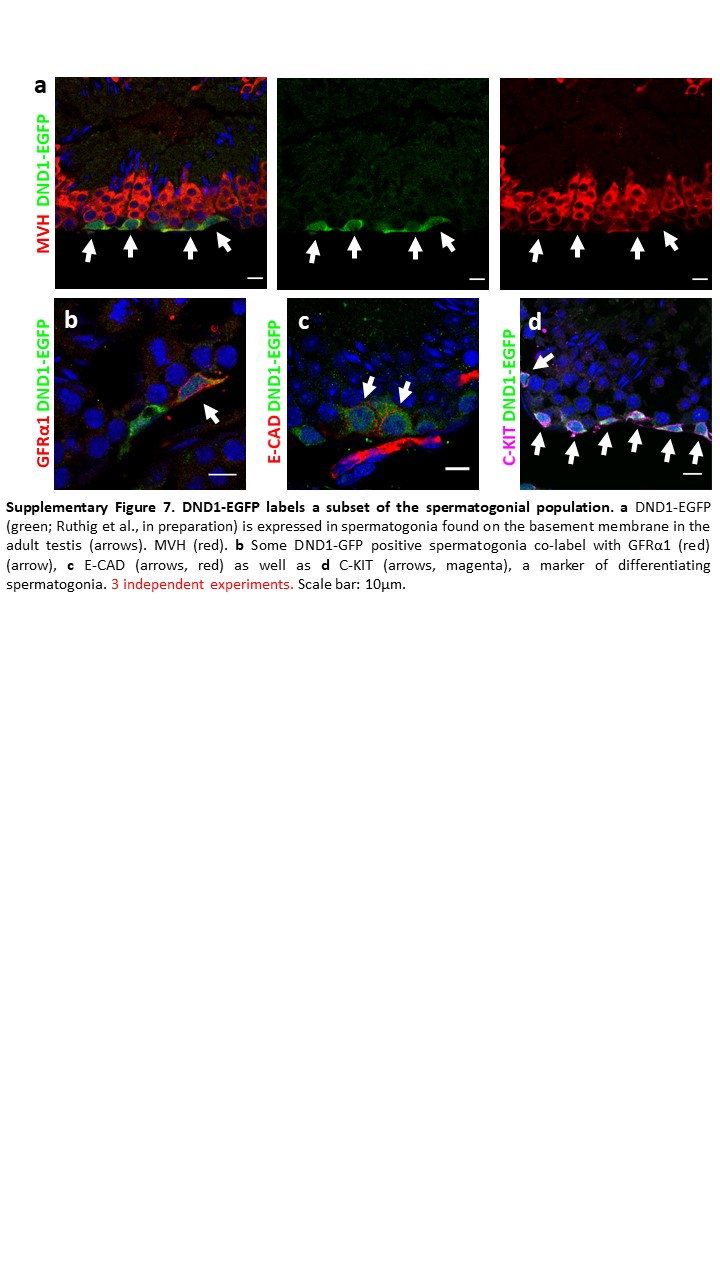


**Supplementary Figure 7. DND1-EGFP labels a subset of the spermatogonial population. a** DND1-EGFP (green; Ruthig et al., in preparation) is expressed in spermatogonia found on the basement membrane in the adult testis (arrows). MVH (red). **b** Some DND1-GFP positive spermatogonia co-label with GFRα1 (red) (arrow), **c** E-CAD (arrows, red) as well as **d** C-KIT (arrows, magenta), a marker of differentiating spermatogonia. 3 independent experiments. Scale bar: 10µm.


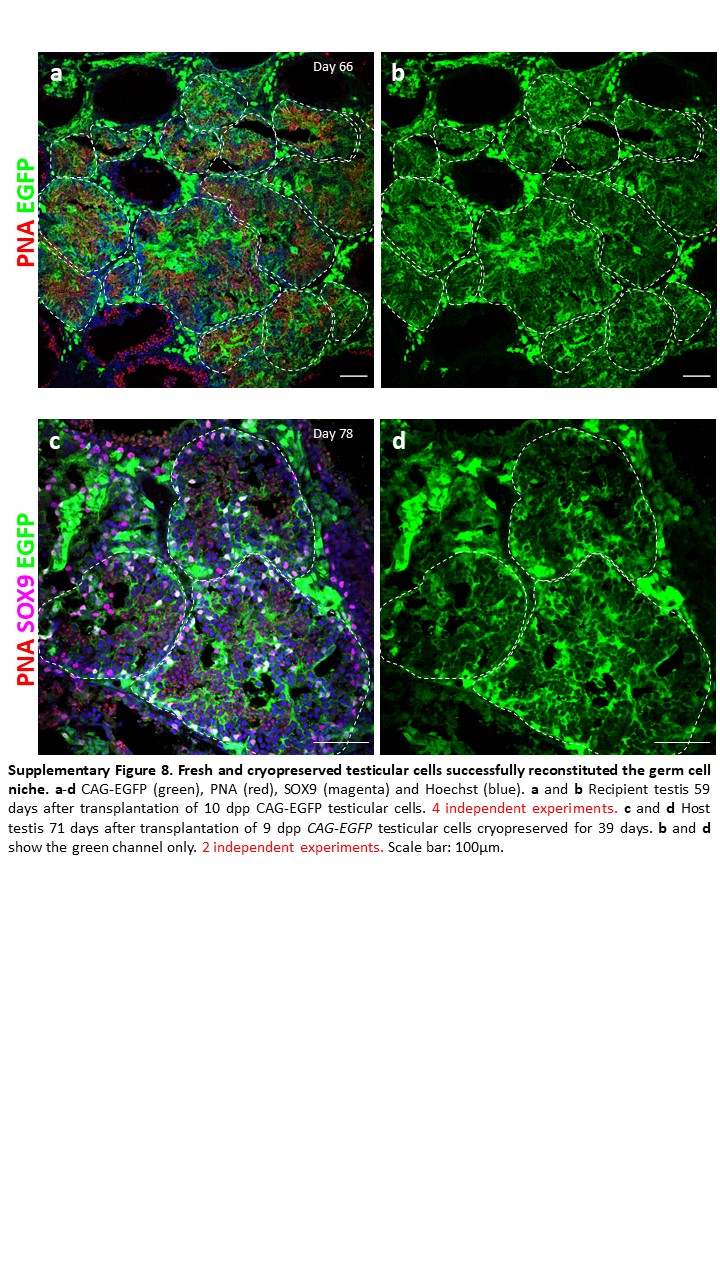


**Supplementary Figure 8. Fresh and cryopreserved testicular cells successfully reconstituted the germ cell niche.** **a** and **b** Recipient testis 59 days after transplantation of 10 dpp CAG-EGFP testicular cells. 4 independent experiments. CAG-EGFP (green), PNA (red) and Hoechst (blue). **c** and **d** Host testis 71 days after transplantation of 9 dpp CAG-EGFP testicular cells cryopreserved for 39 days. 2 independent experiments. CAG-EGFP (green), PNA (red), SOX9 (magenta) and Hoechst (blue). **b** and **d** show the green channel only. Scale bar: 100μm.

Supplementary Table 1. List of primary antibodies used in this study

| **Primary Antibody** | **Host Species** | **Dilution** | **Source** | **Product #** |
| --- | --- | --- | --- | --- |
| AF568-Conjugated Lectin PNA From *Arachis hypogaea* (peanut) | N/A | 1:600 | Thermo Scientific | L32458 |
| AMH/MIS | Goat | 1:500 | Santa Cruz Biotechnology | sc-6886 (discontinued) |
| C-KIT | Goat | 1:100 | Novus Biologicals | AF1356 |
| Cleaved Caspase-3 | Rabbit | 1:500 | Cell Signaling | 9661S |
| Cy3-conjugated anti-α-Smooth Muscle Actin | Mouse | 1:500 | Sigma | C-6198 |
| E-Cad | Rat | 1:500 | Novus Biologicals | 13-1900 |
| F4/80 | Rat | 1:1000 | AbD Serotec | MCA497RT |
| GATA-4 | Mouse | 1:500 | Santa Cruz Biotechnology | sc-25310 |
| GFP | Chicken | 1:1000 | Aves | GFP-1020 |
| GFRα1 | Goat | 1:100 | R&D | AF560 |
| HuC/D | Human | 1:10000 | Gift from V. Lennon  (Mayo Clinic)* | N/A |
| KI67 | Rabbit | 1:500 | Thermo Scientific | RM-9106-S |
| Laminin | Rabbit | 1:500 | Gift from Harold Erickson (Duke University) | N/A |
| MVH/DDX4 | Rabbit | 1:250 | Abcam | ab13840 |
| PECAM1 | Rat | 1:250 | BD Biosciences | 557395 |
| SOX9 | Rabbit | 1:2000 | Millipore | AB5535 |
| SOX9 | Goat | 1:1000 | R&D | AF3075 |
| STRA8 | Rabbit | 1:1000 | Gift from Pierre Chambon  (Institute for Genetics and Cellular and Molecular Biology, Strasbourg, France) | N/A |
| TRA98 | Rat | 1:1000 | Abcam | ab82527 |
| γH2AX | Rabbit | 1:500 | Upstate | 05-636 |
| 3βHSD | Rabbit | 1:100 | TransGenic Inc | KO607 |

* HuC/D / ANNA-1 antisera pooled from 3 patients, first reported in Lennon *et al*, 1991(4)

Supplementary Table 2. List of secondary antibodies used in this study

| **Secondary Antibody** | **Dilution** | **Source** | **Product #** |
| --- | --- | --- | --- |
| AF488 Goat anti-**Rabbit** | 1:500 | Life Technologies | A-11008 |
| AF555 Goat anti-**Rabbit** | 1:500 | Life Technologies | A-21429 |
| AF647 Goat anti-**Rabbit** | 1:500 | Life Technologies | A-21244 |
| Cy3 Donkey anti-**Goat** | 1:1000 | Jackson ImmunoResearch | 705-165-147 |
| AF647 Donkey anti-**Goat** | 1:1000 | Life Technologies | A-21447 |
| AF488 Donkey anti-**Chicken** | 1:1000 | Jackson ImmunoResearch | 703-545-155 |
| Cy5 Donkey anti-**Human** | 1:400 | Jackson ImmunoResearch | 709-175-149 |
| AF647 Goat anti-**Mouse** | 1:500 | Life Technologies | A-21241 |
| AF555 Goat anti-**Rat** | 1:500 | Life Technologies | A-21434 |
| AF488 Donkey anti-**Rat** | 1:1000 | Life Technologies | A-21208 |
