# Supplementary material for: Sertoli cell ablation and replacement of the spermatogonial niche in mouse
Source: Nat Commun. 2020 Jan 2;11:40. doi: 10.1038/s41467-019-13879-8 (PMC6940386; doi:10.1038/s41467-019-13879-8)
Supplement: Supplementary file 2 — Reporting Summary [file 41467_2019_13879_MOESM2_ESM.pdf]

nature research

Corresponding author(s): Blanche Capel

Last updated by author(s): 2019/11/5

Reporting Summary

Nature Research wishes to improve the reproducibility of the work that we publish. This form provides structure for consistency and transparency in reporting. For further information on Nature Research policies, see Authors & Referees and the Editorial Policy Checklist.

Statistics

For all statistical analyses, confirm that the following items are present in the figure legend, table legend, main text, or Methods section.

n/a	Confirmed	
☐	☒	The exact sample size (*n*) for each experimental group/condition, given as a discrete number and unit of measurement
☐	☒	A statement on whether measurements were taken from distinct samples or whether the same sample was measured repeatedly
☐	☒	The statistical test(s) used AND whether they are one- or two-sided<br>*Only common tests should be described solely by name; describe more complex techniques in the Methods section.*
☒	☐	A description of all covariates tested
☒	☐	A description of any assumptions or corrections, such as tests of normality and adjustment for multiple comparisons
☐	☒	A full description of the statistical parameters including central tendency (e.g. means) or other basic estimates (e.g. regression coefficient) AND variation (e.g. standard deviation) or associated estimates of uncertainty (e.g. confidence intervals)
☐	☒	For null hypothesis testing, the test statistic (e.g. F, t, r) with confidence intervals, effect sizes, degrees of freedom and P value noted<br>*Give P values as exact values whenever suitable.*
☒	☐	For Bayesian analysis, information on the choice of priors and Markov chain Monte Carlo settings
☒	☐	For hierarchical and complex designs, identification of the appropriate level for tests and full reporting of outcomes
☒	☐	Estimates of effect sizes (e.g. Cohen's *d*, Pearson's *r*), indicating how they were calculated

Our web collection on statistics for biologists contains articles on many of the points above.

Software and code

Policy information about availability of computer code

Data collection	All images were collected using a Zeiss 780 upright confocal laser scanning, equipped with a ZEN 2010 software.
Data analysis	Immunofluorescent stainings were analyzed with the Fiji version 1.52g The GraphPad Prism version 6 was used for statistical analysis.

For manuscripts utilizing custom algorithms or software that are central to the research but not yet described in published literature, software must be made available to editors/reviewers. We strongly encourage code deposition in a community repository (e.g. GitHub). See the Nature Research guidelines for submitting code & software for further information.

Data

Policy information about availability of data

All manuscripts must include a data availability statement. This statement should provide the following information, where applicable:
- Accession codes, unique identifiers, or web links for publicly available datasets
- A list of figures that have associated raw data
- A description of any restrictions on data availability

All relevant data are available from the corresponding authors on reasonable request.

Field-specific reporting

Please select the one below that is the best fit for your research. If you are not sure, read the appropriate sections before making your selection.

☒ Life sciences ☐ Behavioural & social sciences ☐ Ecological, evolutionary & environmental sciences

For a reference copy of the document with all sections, see nature.com/documents/nr-reporting-summary-flat.pdf

Life sciences study design

All studies must disclose on these points even when the disclosure is negative.

Sample size	Not effect size were predetermined. Sample size was determined according to previous experience in the field, to detect differences that are statistically significant.
Data exclusions	No data was excluded from the analyses.
Replication	All experiments were reproduced at least three times with independent biological samples, except for isolated Sertoli transplantation experiments (Figure 3), canine testis culture experiment (Supplementary Figure 5), isolation of SOX9-ECFP Sertoli cell analysis (Supplementary Figure 6) and cryopreserved testicular cell transplantation study (Figure 5 and Supplementary Figure 8) that were repeated twice with more than 2 independent animals.
Randomization	No method of randomization was used as in mice one testis was for control and the other for treated group.
Blinding	Blinding was not possible.

Reporting for specific materials, systems and methods

We require information from authors about some types of materials, experimental systems and methods used in many studies. Here, indicate whether each material, system or method listed is relevant to your study. If you are not sure if a list item applies to your research, read the appropriate section before selecting a response.

Materials & experimental systems

n/a	Involved in the study	
☐	☒	Antibodies
☒	☐	Eukaryotic cell lines
☒	☐	Palaeontology
☐	☒	Animals and other organisms
☒	☐	Human research participants
☒	☐	Clinical data

Methods

n/a	Involved in the study	
☒	☐	ChIP-seq
☐	☒	Flow cytometry
☒	☐	MRI-based neuroimaging

Antibodies

Antibodies used

Primary antibodies:
AF568-Conjugated Lectin PNA From Arachis hypogaea (peanut) (Thermo Scientific, cat no. L32458, lot no. L32458)
Anti-AMH/MIS (Santa Cruz Biotechnology, cat no. sc-6886 (discontinued), lot no. H0516)
Anti-C-KIT (Novus Biologicals, cat no. AF1356, lot no. IEO0217011)
Anti-Cleaved Caspase-3 (Cell Signaling, cat no. 9661S, lot no. 43)
Cy3-conjugated anti-α-Smooth Muscle Actin (Sigma, cat no. C-6198, lot no. 127M4859V)
Anti-E-Cad (Novus Biologicals, cat no. 13-1900, lot no. UB2682693)
Anti-F4/80 (AbD Serotec, cat no. MCA497RT, lot no. 1003)
Anti-GATA-4 (Santa Cruz Biotechnology, cat no. sc-25310, lot no. C1417)
Anti-GFP (Aves, cat no. GFP-1020, lot no. GFP879484)
Anti-GFRα1 (R&D, cat no. AF560)
Anti-HuC/D (gifted from V. Lennon)
Anti-KI67 (Thermo Scientific, cat no. RM-9106-S, lot no. 06S1607L)
Anti-Laminin (gifted from Harold Erickson)
Anti-MVH/DDX4 (Abcam, cat no. ab13840, lot no. GR3245728-1)
Anti-PECAM1(BD Biosciences, cat no. 557395, lot no. 8043575)
Anti-SOX9 (Millipore, cat no. AB5535, lot no. 3107073)
Anti-SOX9 (R&D, cat no. cat no. AF3075, lot no. WIL0417051)
Anti-STRA8 (gifted from Pierre Chambon)
Anti-TRA98 (Abcam, cat no. ab82527, lot no. GR3183423-7)
Anti-γH2AX (Upstate, cat no. 05-636, lot no. 23646)
Anti-3βHSD (TransGenic Inc, cat no. KO607, lot no. TG030317)

Secondary antibodies:
AF488 Goat anti-Rabbit (Life Technologies, cat no. A-11008, lot no. 44894A)
AF555 Goat anti-Rabbit (Life Technologies, cat no. A-21429, lot no. 2015563)
AF647 Goat anti-Rabbit (Life Technologies, cat no. A-21244, lot no. 1783430)
Cy3 Donkey anti-Goat (Jackson ImmunoResearch, cat no. 705-165-147, lot no. 139052)
AF647 Donkey anti-Goat (Life Technologies, cat no. A-21447, lot no. 2045332)
AF488 Donkey anti-Chicken (Jackson ImmunoResearch, cat no. 703-545-155, lot no. 140640)
Cy5 Donkey anti-Human (Jackson ImmunoResearch, cat no. 709-175-149, lot no. 134761)

AF647 Goat anti-Mouse (Life Technologies, cat no. A-21241, lot no. 1889313)
AF555 Goat anti-Rat (Life Technologies, cat no. A-21434, lot no. 1008806)
AF488 Donkey anti-Rat (Life Technologies, cat no. A-21208, lot no. 2032378)

Detailed of all antibodies are included within the text or within Supplemental Table 1.

Validation

>AF568-Conjugated Lectin PNA From Arachis hypogaea (peanut) (Thermo Scientific, cat no. L32458, lot no. L32458)
Validation conducted by providing company.
>Anti-AMH/MIS (Santa Cruz Biotechnology, cat no. sc-6886 (discontinued), lot no. H0516)
Neural crest-derived neurons invade the ovary but not the testis during mouse gonad development. McKey J et al. PNAS. 2019.
>Anti-C-KIT (Novus Biologicals, cat no. AF1356, lot no. IEO0217011)
Macrophages contribute to the spermatogonial niche in the adult testis. DeFalco T et al. Cell Rep. 2015.
>Anti-Cleaved Caspase-3 (Cell Signaling, cat no. 9661S, lot no. 43)
Numb regulates somatic cell lineage commitment during early gonadogenesis in mice. Lin YT et al. Development 2017.
>Cy3-conjugated anti-α-Smooth Muscle Actin (Sigma, cat no. C-6198, lot no. 127M4859V)
Defective remodeling and maturation of the lymphatic vasculature in Angiopoietin-2 deficient mice. Dellinger M et al. Dev. Biol. 2008.
>Anti-E-Cad (Novus Biologicals, cat no. 13-1900, lot no. UB2682693)
Numb regulates somatic cell lineage commitment during early gonadogenesis in mice. Lin YT et al. Development. 2017.
>Anti-F4/80 (AbD Serotec, cat no. MCA497RT, lot no. 1003)
Macrophages contribute to the spermatogonial niche in the adult testis. DeFalco T et al. Cell Rep. 2015.
>Anti-GATA-4 (Santa Cruz Biotechnology, cat no. sc-25310, lot no. C1417)
Validation conducted by providing company.
>Anti-GFP (Aves, cat no. GFP-1020, lot no. GFP879484)
Numb regulates somatic cell lineage commitment during early gonadogenesis in mice. Lin YT et al. Development 2017.
>Anti-GFRα1 (R&D, cat no. AF560)
Transcription factor GLIS3: a new and critical regulator of postnatal stages of mouse spermatogenesis. Kang HS et al. Stem Cells. 2016.
>Anti-HuC/D (gifted from V. Lennon)
Neural crest-derived neurons invade the ovary but not the testis during mouse gonad development. McKey J et al. PNAS. 2019.
>Anti-KI67 (Thermo Scientific, cat no. RM-9106-S, lot no. 06S1607L)
Macrophages contribute to the spermatogonial niche in the adult testis. DeFalco T et al. Cell Rep. 2015.
>Anti-Laminin (gifted from Harold Erickson)
Numb regulates somatic cell lineage commitment during early gonadogenesis in mice. Lin YT et al. Development. 2017.
>Anti-MVH/DDX4 (Abcam, cat no. ab13840, lot no. GR3245728-1)
Neural crest-derived neurons invade the ovary but not the testis during mouse gonad development. McKey J et al. PNAS. 2019.
>Anti-PECAM1(BD Biosciences, cat no. 557395, lot no. 8043575)
Numb regulates somatic cell lineage commitment during early gonadogenesis in mice. Lin YT et al. Development. 2017.
>Anti-SOX9 (Millipore, cat no. AB5535, lot no. 3107073)
Numb regulates somatic cell lineage commitment during early gonadogenesis in mice. Lin YT et al. Development. 2017.
>Anti-SOX9 (R&D, cat no. cat no. AF3075, lot no. WIL0417051)
Validation conducted by providing company.
>Anti-STRA8 (gifted from Pierre Chambon)
Left-Biased spermatogenic failure in 129/SvJ Dnd1Ter/+ mice correlates with differences in vascular architecture, oxygen availability, and metabolites. Bustamante-Marin XM et al. BOR. 2015.
>Anti-TRA98 (Abcam, cat no. ab82527, lot no. GR3183423-7)
Validation conducted by providing company.
>Anti-γH2AX (Upstate, cat no. 05-636, lot no. 23646)
Left-Biased spermatogenic failure in 129/SvJ Dnd1Ter/+ mice correlates with differences in vascular architecture, oxygen availability, and metabolites. Bustamante-Marin XM et al. BOR. 2015.
>Anti-3βHSD (TransGenic Inc, cat no. KO607, lot no. TG030317)
In vitro reconstruction of mouse seminiferous tubules supporting germ cell differentiation. Yokonishi T et al. BOR. 2013.

Animals and other organisms

Policy information about studies involving animals; ARRIVE guidelines recommended for reporting animal research

Laboratory animals

Mouse:
8 to 10-week-old CD1 (from Charles River Laboratories) females and males.
8 to 10-week-old C57BL/6J (from The Jackson Laboratories) females and males.
8 to 10-week-old FVB (from The Jackson Laboratories) females and males.
8 to 10-week-old F1 (FVB x C57BL/6J) males.
8 to 10-week-old FVB CAG-EGFP (Ref. 33) females and males.
8 to 10-week-old Sox9 (Sry-box 9)-ECFP (Ref. 34) females and males.
2 to 8-weeks old Dnd1(dead end homolog 1)-EGFP (Ruthig et al., in preparation) males.
In transplantation studies, Sox9-ECFP or CAG-EGFP donors were obtained from homozygous breeding lines and used between 6.5-10.5 dpp.

Wild animals

Our study did not involve wild animals.

Field-collected samples

Our study did not involve Field-collected samples.

Ethics oversight

This study was performed under in accordance with protocols approved by Duke University Institutional Animal Welfare and Use

Ethics oversight | Committee.

Note that full information on the approval of the study protocol must also be provided in the manuscript.

Flow Cytometry

Plots

Confirm that:

☒ The axis labels state the marker and fluorochrome used (e.g. CD4-FITC).

☒ The axis scales are clearly visible. Include numbers along axes only for bottom left plot of group (a 'group' is an analysis of identical markers).

☒ All plots are contour plots with outliers or pseudocolor plots.

☒ A numerical value for number of cells or percentage (with statistics) is provided.

Methodology

Sample preparation	Sample preparation was described in the manuscript.
Instrument	MoFlo Astrios EQ
Software	Summit V6.3.1.16945
Cell population abundance	The sorted cells were reanalyzed and the % GFP+ cells out of cells was determined 91% (Supplemental Figure 6).
Gating strategy	The GFP positive gate was set based on wild type testis vs GFP expressing cells.

☒ Tick this box to confirm that a figure exemplifying the gating strategy is provided in the Supplementary Information.
